# Therapeutic approach of Lyell syndrome with infliximab and dexamethasone pulse: report of a clinical case^☆^

**DOI:** 10.1016/j.abd.2020.10.020

**Published:** 2022-04-02

**Authors:** Jânia Dara Jácome Pacheco, Maria Rodrigues Viegas Ribeiro, Catarina Sousa Duque Soares Queirós, Maria Fátima Cameira Martins Xambre

**Affiliations:** a Department of Anaesthesiology, Hospital de Santa Maria, Centro Hospitalar Universitário Lisboa Norte, Lisboa, Portugal; b Department of Dermatology, Hospital de Santa Maria, Centro Hospitalar Universitário Lisboa Norte, Lisboa, Portugal

Dear Editor,

Toxic Epidermal Necrolysis (TEN) is a rare, life-threatening mucocutaneous disease.

A 23-year-old female presented with TEN after treatment with diclofenac, metamizole and etoricoxib. She was febrile, with oral, ocular, and perineal mucositis and a maculopapular rash involving an estimated body surface area of 20%. She was admitted to the dermatology department, skin biopsies were collected, and Intravenous Immunoglobulin (IVIg) was started. However, her clinical condition worsened, so she was transferred to the burn unit.

Upon admission, the patient had an extensive maculopapular rash. There were also positive Nikolsky signs in her face, back, palms and feet soles, and involvement of the oral, ocular, vaginal and perineal mucous membranes (Figure 1, Figure 2). Additionally, given the upper airway edema, she was orotracheal intubated.

**Figure 1:**
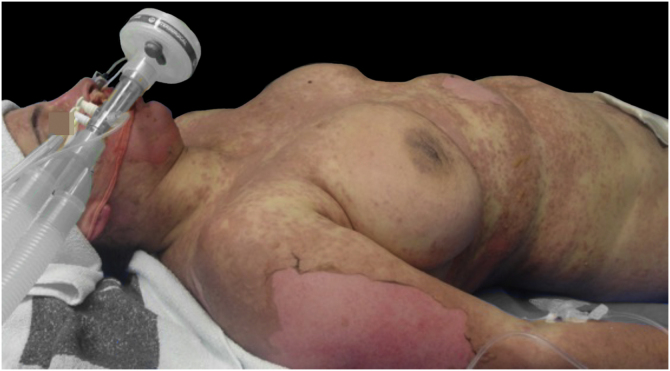
Blisters, vesicles, and detachment of the epidermis.

**Figure 2:**
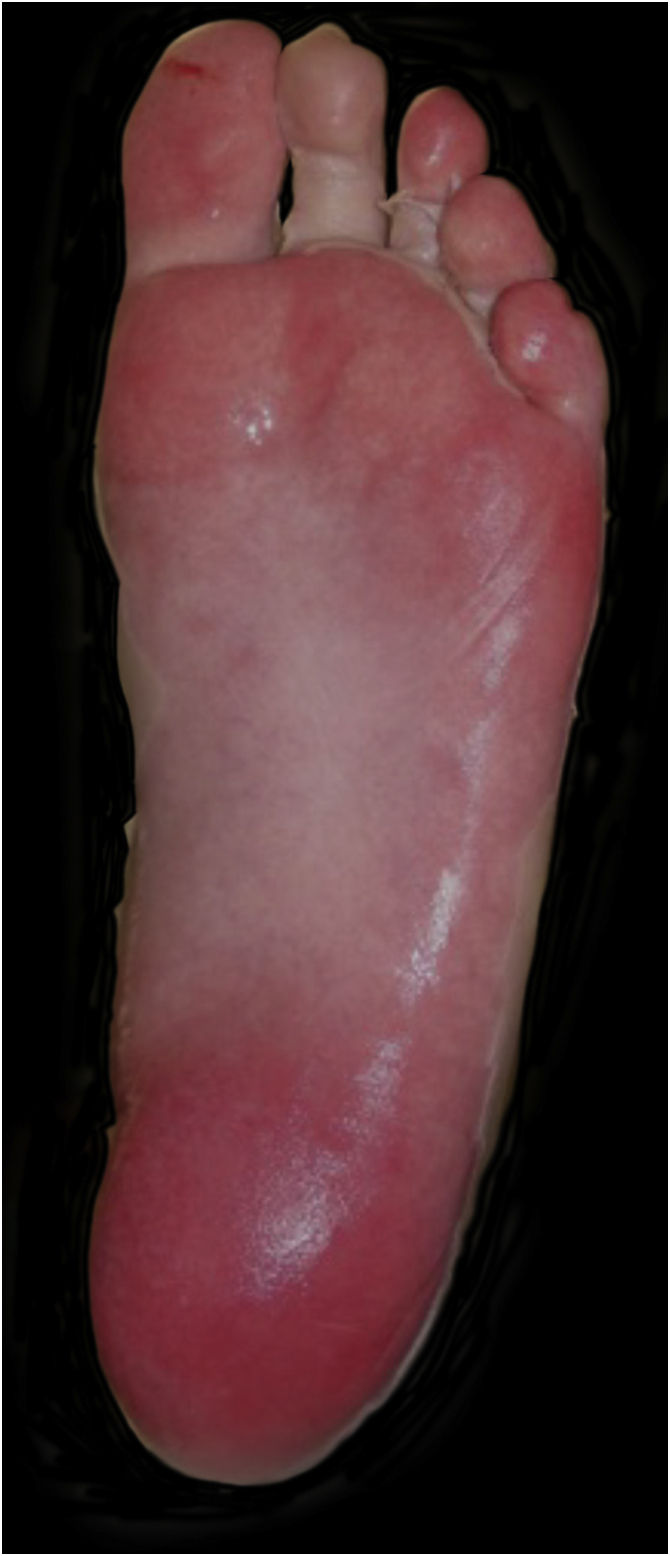
Detachment of the epidermis.

Under immunoglobulin therapy, there was a clinical worsening, with an increase in the body surface involvement, from 85% to 100%. In addition, she developed multiorgan failure with hematological, cardiovascular, hepatic, gastrointestinal and renal dysfunction.

On the eighth day of hospitalization, the patient received a single infliximab dose (5 mg/kg, EV) and was started on a pulse of dexamethasone (100 mg, EV × 1/day) for three days. After administration of infliximab and dexamethasone pulse, the patient evolved favorably with the progressive resolution of multiorgan dysfunction, and on the nineteenth day, the patient presented epithelialization of the entire body surface.

According to literature, TEN is a severe idiosyncratic reaction, mostly drug-induced. It is characterized by a detachment of the skin and mucous membranes at the level of the dermis-epidermis junction as a result of the necrosis of keratinocytes.

The diagnosis of TEN is clinical. It is characterized by a prodromal phase with flu-like symptoms that precede the appearance of mucocutaneous lesions. The confirmation of the diagnosis involves a skin biopsy.^1^

In patients with probable drug-induced TEN, early diagnosis and immediate removal of the drug are essential to improve the prognosis.

Apart from supportive care, there is no established treatment. However, several immunosuppressants and immunomodulators have been used, and although there are no adequate randomized studies, some drugs have shown promise.

Corticosteroids were among the first drugs used; however, their use is currently controversial. Although some studies demonstrate a worse prognosis,^2^ there are others that show benefits with the administration of high doses over a short period of time.^3^

IVIg used to be the most consensual therapy for the treatment of this syndrome. However, a meta-analysis published in 2012 concluded that this drug is not associated with a better prognosis.^4^

TNF-alpha inhibitors seem promising drugs in the treatment of TEN. There are some case reports where a single dose of infliximab or etanercept interrupted the progression of the disease and induced regression of the skin lesions.^5^

In fact, the case we reported is in line with these findings since the patient did not show any improvement after IVIg administration but had a great response to infliximab and high-dose dexamethasone.

## Financial support

None declared.

## Authors' contributions

Jânia Dara Jácome Pacheco: Collection, analysis, and interpretation of data; effective participation in research orientation; intellectual participation in the propaedeutic and/or therapeutic conduct of the studied cases; management of case studies; critical review of the manuscript; drafting and editing of the manuscript; critical review of the literature.

Maria Rodrigues Viegas Ribeiro: Critical review of the literature; collection, analysis, and interpretation of data; drafting and editing of the manuscript.

Catarina Sousa Duque Soares Queirós: Critical review of the manuscript.

Maria Fátima Cameira Martins Xambre: Approval of the final version of the manuscript.

## Conflicts of interest

None declared.
